# Machine Learning Algorithms for Predicting Psychostimulant Intoxication in Emergency Department Patients: A Multicenter Study

**DOI:** 10.5811/westjem.63917

**Published:** 2026-10-18

**Authors:** Jiun-Hao Yu, Yueh-Peng Chen, Chih-Huang Hsieh, Yu-Jang Su, Hsien-Yi Chen, Chih-Chuan Lin, Te-I Weng, Cheng-Chung Fang, Sung-Wei Liu, Yen-Chia Chen, Yu-Cheng Pei, Kuan-Fu Chen

**Affiliations:** * China Medical University Hsinchu Hospital, Department of Emergency Medicine, Hsinchu, Taiwan; † Chang Gung University, Graduate Institute of Management, Taoyuan City, Taiwan; ‡ Yuanpei University of Medical Technology, Hsinchu, Taiwan; § Chang Gung University, Master of Science Degree Program in Innovation for Smart Medicine, Taoyuan, Taiwan; || Chang Gung Medical Technology Co Ltd, Department of Artificial Intelligence Research and Development, Taoyuan, Taiwan; # MacKay Memorial Hospital, Toxicology Division and Poison Center, Taipei, Taiwan; ¶ Chang Gung Memorial Hospital, Department of Emergency Medicine, LinKou, Taiwan; ** Chang Gung University, College of Medicine, Taoyuan, Taiwan; †† National Taiwan University, College of Medicine, Institute of Forensic Medicine, Taipei, Taiwan; ‡‡ National Taiwan University, College of Medicine and National Taiwan University Hospital, Department of Emergency Medicine, Taipei, Taiwan; §§ National Taiwan University, College of Medicine and National Taiwan University Hospital, Forensic and Clinical Toxicology Center, Taipei, Taiwan; |||| Buddhist Tzu Chi Medical Foundation and Tzu Chi University, Department of Emergency, Hualien Tzu Chi Hospital, Hualien, Taiwan; ## Tzu Chi University, Institute of Medical Sciences, Hualien, Taiwan; ¶¶ Taipei Veterans General Hospital, Emergency Department, Taipei, Taiwan; 15 National Yang Ming Chiao Tung University, School of Medicine, Department of Emergency Medicine, Taipei, Taiwan; 16 Chang Gung Memorial Hospital, Department of Physical Medicine and Rehabilitation, LinKou, Taoyuan City, Taiwan; 17 Chang Gung Memorial University, Master of Science Degree Program in Innovation for Smart Medicine, Taoyuan City, Taiwan; 18 Chang Gung University, School of Medicine, College of Medicine, Taoyuan City, Taiwan; 19 Chang Gung Memorial Hospital at LinKou, Center for Vascularized Composite Allotransplantation, Taoyuan City, Taiwan; 20 Chang Gung University, Clinical Informatics and Medical Statistics Research Center, Taoyuan, Taiwan; 21 Chang Gung University, Research Center for Emerging Viral Infections, Taoyuan, Taiwan; 22 Chang Gung Memorial Hospital, Department of Emergency Medicine, Keelung, Taiwan; 23 Chang Gung University, Department of Artificial Intelligence, College of Intelligent Computing, Taoyuan, Taiwan

## Abstract

**Introduction:**

Psychostimulant detection rates among emergency department (ED) patients presenting with suspected substance intoxication are low and are influenced by limited access to confirmatory testing, insufficient clinical information, and variability in physician experience. These challenges often result in high false-positive rates and unnecessary resource use. We hypothesized that machine learning (ML) algorithms could maintain reliable diagnostic performance across a temporal validation cohort and perform comparably to physicians with varying experience levels.

**Methods:**

This multicenter study prospectively enrolled ED patients with suspected substance intoxication between March 2019 and January 2020. We chronologically partitioned data into a training set (March–October 2019) and a temporal validation cohort (November 2019–January 2020) to assess model generalizability. After determining psychostimulant intoxication via liquid chromatography-mass spectrometry from urine samples, we trained the ML algorithms using the confirmed positive cases. The primary outcome was the diagnostic discrimination of the models, measured by the area under the receiver operating characteristic curve (AUC) in the temporal validation cohort. Secondary outcomes included model accuracy, the Youden index, and a comparative assessment of sensitivity and specificity relative to five physicians of varying experience levels.

**Results:**

Among 1,382 patients (mean age 43.8 [standard deviation 19.2] years; 55.3% male), 302 patients (21.9%) had confirmed psychostimulant intoxication, primarily involving (meth)amphetamines (134 [44.4%]), cathinones (107 [35.4%]), and ketamine (97 [32.1%]). In the temporal validation cohort (*n* = 382), the ML models demonstrated consistent performance, with AUCs ranging from 0.786 to 0.836. The primary outcome was met by the top-performing extreme gradient boosting (XGB) model (AUC, 0.836, 95% CI, 0.785–0.882; accuracy, 0.866 (95% CI, 0.838–0.895); and Youden index, 0.590 (95% CI, 0.480–0.694). To evaluate secondary outcomes, the XGB model’s predictive accuracy was evaluated against 5 emergency physicians. The model surpassed the junior emergency physicians in both specificity (0.910 vs 0.633–0.867) and sensitivity (0.681 vs 0.500–0.633); its specificity was comparable to that of the senior emergency physicians (0.900–0.933).

**Conclusion:**

Machine learning models provide reliable diagnostic utility for identifying psychostimulant intoxication, achieving performance comparable to senior emergency physicians. While moderate sensitivity (0.389–0.708) limits their role as standalone rule-out tools, their high specificity provides a standardized tool to assist clinical decision-making and reduce diagnostic uncertainty in the fast-paced ED setting.

## INTRODUCTION

The global increase in the use of both traditional and new psychoactive substances poses significant public health concerns.^1^ Many of these substances are psychoactive stimulants that consist of diverse compounds, which can induce various effects other than sympathomimetic toxidromes^2^ and are often designed to elude standard toxicological screens.^3^ While commonly used screening tools such as enzyme-linked immunosorbent assays (ELISA) may exhibit cross-reactivity among a range of synthetic compounds,^4^,^5^ the gold standard test, liquid chromatography-mass spectrometry (LC-MS/MS), often requires several days for results and is only available in select hospitals.

Current symptom-based approaches for identifying psychostimulant intoxication often result in high false-positive rates, as evidenced by the Taiwan Emergency Department Drug Abuse Surveillance (TEDAS) project, which reported a substance detection rate of only 25.5% via LC-MS/MS.^6^ Clinical information that physicians rely on to identify cases, such as patient history, symptoms, and initial laboratory results, can be used as input for a machine learning (ML) algorithm to deliver real-time predictions. By ruling out most negative cases, the algorithm could minimize unnecessary confirmatory testing and expert consultations, allowing emergency department (ED) resources to focus on high-likelihood patients. This integration may enhance diagnostic precision, optimize ED efficiency, and support less experienced physicians in managing suspected cases of psychostimulant intoxication.

Machine learning can address these challenges by analyzing large datasets, recognizing complex patterns,^7^ and identifying cases that might be missed by physicians.^8^ This approach can trigger alarms for suspected cases, enabling physicians to consult with toxicologists and conduct confirmatory testing. The datasets used to train ML models often integrate domain expertise,^9^,^10^ enriching the training data with expert insights to increase the accuracy of ML algorithms. Consequently, these algorithms can serve as valuable tools to assist frontline physicians in identifying suspected cases of psychostimulant intoxication. Prior toxicology research has primarily applied ML for exploring the structures of new psychoactive substances via LC-MS/MS spectra,^11^ recognizing toxidromes based solely on physical examination findings^8^ and predicting risk and stratification related to opioid overdose.^12^ However, data on the use of ML for predicting psychostimulant toxicity are lacking, highlighting an area for further study.

We hypothesized that ML algorithms could serve as a robust decision-support tool for identifying psychostimulant intoxication during initial ED evaluations. Specifically, we predicted that these algorithms would maintain reliable diagnostic performance across a temporal validation cohort and demonstrate superior overall accuracy and specificity compared to emergency physicians of varying experience levels, thereby mitigating diagnostic anchoring in fast-paced clinical environments.

**Population Health Research Capsule**

What do we already know about this issue?

*Psychostimulant intoxication is difficult to recognize in the emergency department (ED) due to overlap with many medical and psychiatric conditions.*

What was the research question?

*Can machine learning reliably identify psychostimulant intoxication in the ED?*

What was the major finding of the study?

*Extreme gradient boosting achieved an area under the curve of 0.836 (95% CI, 0.785–0.882) with 91.0% specificity.*

How does this improve population health?

*Highly specific machine learning algorithms offer a standardized diagnostic benchmark, reducing clinical variability and optimizing resource use.*

## METHODS

### Study Design and Setting

This prospective study was conducted in the EDs of 10 hospitals across six major medical systems in Taiwan from March 2019 to January 2020, with study protocols approved by the institutional review boards of each system: China Medical University Hospitals (CMUH110-REC3-038); Chang Gung Memorial Hospitals (202001418B0); National Taiwan University Hospitals (201711102RIND); Hualien Tzu Chi Hospitals (109-165-B); Taipei Veterans General Hospitals (2018-07-049BC); and MacKay Memorial Hospitals (20MMHIS036e). The study settings comprise six academic tertiary medical centers and four major designated emergency responsibility hospitals. Because Taiwan lacks a mandatory referral system, these high-volume centers manage a diverse patient population, capturing a broad spectrum of clinical severity ranging from mild cases to critical, high-acuity emergencies.

The participating hospitals were part of the TEDAS network, a nationwide program to monitor recreational drug use among ED patients via qualitative LC-MS/MS analysis to detect 110 illicit substances in urine samples (Supplementary Table S1).^6^ Substances with detected concentrations less than 50 ng/mL are reported as negative. This cutoff enhances the sensitivity for detecting both predominant and emerging substances in polysubstance use patterns, including subtle substances and clinically significant metabolites such as those of marijuana. In this study, we adopted a broad definition of psychostimulants to include both classical sympathomimetics and substances such as synthetic cannabinoids, phencyclidine-type substances, tryptamines, and marijuana, which may exhibit sympathomimetic-like effects through complex mechanisms of action. Accordingly, of the 110 substances analyzed, 104 are classified as psychostimulants, including categories such as synthetic cannabinoids, phenethylamines, synthetic cathinones, phencyclidine-type substances, tryptamines, piperazines, amphetamine-types, marijuana, cocaine, and others. The remaining 6 substances—etizolam, phenazepam, diclazepam, codeine, morphine, and 6-acetylmorphine—were excluded because they are not classified as psychostimulants. Anonymized urine samples from all enrolled patients were sent to two national laboratories for LC-MS/MS analysis. The need for individual informed consent was waived by the Taiwan Food and Drug Administration (Ministry of Health and Welfare document No. 1010265079).

### Selection of Participants

We prospectively enrolled patients with suspected substance ingestion based on 1 or more of the following criteria: bizarre or violent behavior; self-harm; sympathomimetic toxidromes (such as agitation, hyperthermia, tachycardia, and hypertension); altered mental status; first seizure without an apparent cause; reported substance use, traumatic events paired with a clinical suspicion of substance use, or uncertain cause of cardiac arrest in patients <65 years of age. We excluded from this study patients who declined the urine LC-MS/MS analysis or whose urine samples could not be successfully collected for analysis.

### Data Collection, Preprocessing, and Feature Selection

The inclusion criteria, medical history, clinical symptoms, and vital signs were extracted from standardized, structured medical records completed by primary care physicians at enrollment across all participating hospitals. A trained researcher subsequently reviewed the records to ensure data accuracy and corrected them as necessary. To ensure consistency across sites, the data from each hospital were double-checked by a second researcher. Both the primary care physicians and researchers were blinded to the study hypothesis.

We standardized clinical variables to the initial ED encounter, using triage vital signs and initial clinical assessments. While most laboratory results reflect the first blood draw, peak values were used specifically for creatine kinase, troponin I, and lactate. To manage missing data based on clinical workflow logic, we applied a multitiered strategy. Variables with high missingness (greater. than 50% missing) were processed via dummy coding (one-hot encoding) into three states (positive, negative, and not performed) to preserve informative missingness.^13^ Conversely, structured electronic sheet items (eg, clinical symptoms and medical history) were inherently binary by design; thus, data not marked as positive were considered negative. Continuous variables underwent mean imputation to maintain model stability. All data processing methods are explicitly denoted in Table 1 using symbolic notation to ensure transparency.

To address potential bias from imbalanced datasets, we applied the synthetic minority oversampling technique (SMOTE), which generates synthetic examples of the minority class to improve class distribution and enhance minority class prediction.^14^,^15^ All variables detailed in Table 1, with the exception of ED disposition, were incorporated as features in our ML models. These features included patient demographics, underlying comorbidities, clinical presentation, physical examination findings, and laboratory results.

We identified predictive features for psychostimulant intoxication using the Boruta algorithm, enhancing model accuracy by retaining only the most significant features. By iteratively comparing actual data against generated shadow features, we deemed variables that consistently outperformed their shadow counterparts as relevant and excluded the rest. Finally, we used Shapley additive explanations (SHAP) analysis to interpret the final models, visually representing the clinical impact of each selected feature.^16^

### Modeling Machine Learning Algorithms

We adopted a temporal validation approach, assigning patients enrolled between March 2019 and October 2019 to the training set and those enrolled between November 2019 and January 2020 to the testing set. Subsequently, a 5-fold cross-validation protocol was performed exclusively on the training set to ensure model robustness.

To predict psychostimulant intoxication, we employed five ML techniques: extreme gradient boosting (XGB); gradient boosting machine (GBM); linear discriminant analysis (LDA); multivariable logistic regression (MLR); and multilayer perceptron (MLP). The XGB and GBM are decision tree-based methods known for their parameter tuning flexibility and ability to model complex feature interactions. The LDA and MLR are classical models well-suited for linear data relationships. The MLPs, artificial neural networks, are particularly effective at capturing complex, nonlinear relationships and handling high-dimensional data.

### Outcome Measures

The ML algorithm produced a binary output, classifying patients into psychostimulant intoxication or nonintoxication groups on the basis of the LC-MS/MS results. The primary outcome was the diagnostic discrimination of the ML models, measured by the area under the receiver operating characteristic curve (AUC) when validated against the temporal test set. Secondary outcomes included model accuracy and the Youden index. Additionally, we conducted a comparative performance assessment of the ML algorithms relative to five physicians with varying experience levels to evaluate whether the models achieved comparable or superior specificity and sensitivity to junior emergency physicians. We also calculated the following metrics for all models: positive predictive value (PPV); negative predictive value (NPV); area under the precision-recall curve (PRC); and F1 score. We implemented the ML algorithms using Python, version 3.8.17 (Python Software Foundation) and the Scikit-learn library, version 1.2.2. (Scikit-learn Developers).

### Physician Performance Evaluation

To evaluate physician performance, we used stratified random sampling to extract a subset of 60 patients (30 positive and 30 negative) from the testing database. Five physicians participated: one toxicologist, two senior emergency physicians, and two junior emergency physicians. The toxicologist was certified by the Taiwan Society of Emergency Medicine. Senior physicians had over five years of experience as attending physicians, whereas junior physicians had less than five years. Each physician independently reviewed the patients’ records and categorized them into either the psychostimulant intoxication group or the nonintoxication group.

## RESULTS

### Patient Profile and Baseline Demographics

Between March 2019 and January 2020, we enrolled 1,384 patients. After excluding two subjects due to duplicated registration, the final sample included 1,382 patients (Figure 1). Among these, 370 (26.8%) tested positive for substances via LC-MS/MS. Specifically, 302 patients (21.9%) tested positive for psychostimulants, constituting the psychostimulant intoxication group. The remaining 1,080 patients formed the non-intoxication group. For model development, the cohort was partitioned into a training dataset (*n* = 1,000) and a testing dataset (*n* = 382). The prevalence of psychostimulant positivity remained consistent across both subsets, with 230 (23.0%) in the training set and 72 (18.8%) in the testing set.

Our demographic analysis (Table 1) revealed that the intoxication group was younger than the nonintoxication group (37.0 [14.0] vs 45.7 [20.0] years) and had a higher proportion of males (63.9% vs 53.0%). At triage, the intoxication group presented a faster heart rate than the nonintoxication group (106.8 [27.3] vs 96.4 [24.3] beats per minute). Upon clinical assessment, the intoxication group had higher rates of excited mental states (41.1% vs 17.6%), abnormal behavior or speech (41.1% vs 19.7%), hallucinations (21.9% vs 5.9%), agitation (41.1% vs 17.7%), palpitations (6.3% vs 3.2%), and self-reported substance use (31.8% vs 8.1%). The intoxication group more frequently presented with mild Glasgow Coma Scale (GCS) scores (13–14), whereas severe neurological impairment (GCS 3–8) was more prevalent in the nonintoxication group. Although not statistically significant, the intoxication group had a greater prevalence of dilated pupils (>5 mm) and hyperactive bowel sounds. Brain computed tomography (CT) revealed a lower incidence of intracranial hemorrhage in the intoxication group. Laboratory results revealed elevated white blood cells, hematocrit, and platelets in the intoxication group, whereas elevated creatine kinase levels were more common in the nonintoxication group (26.5% vs 12.6%). Initial screening via urine immunoassay demonstrated false-positive rates for amphetamine (2.8%) and ketamine (1.3%).

### Categories and Combination Patterns of Substances

The substances most frequently detected by LC-MS/MS were amphetamine and methamphetamine, followed by synthetic cathinone, opioids, and ketamine (Supplementary Figure S1). Among the psychostimulant-positive subjects, half (155/302) had mixed substance exposure (Supplementary Table S2). Additionally, 50% of those who tested positive for traditional psychostimulants tested positive for new psychoactive substances (74/142). Seven patients tested positive for benzodiazepines, including etizolam, phenazepam, and diclazepam, all of whom had co-ingested with psychostimulants.

### Prediction Performance of Machine Learning Algorithms and Primary Outcome

The models demonstrated strong temporal stability across distinct cohorts. Most algorithms exhibited robust discriminatory power during training (Figure 2A) and successfully generalized to the validation cohort, maintaining consistent AUCs above 0.820 (Figure 2B). While the MLP demonstrated a comparatively lower baseline performance, it displayed remarkable consistency between phases. As shown in Table 2, the XGB model met the primary outcome as the top-performing algorithm, achieving an AUC of 0.836 (95% CI, 0.785–0.882), an accuracy of 0.866 (95% CI, 0.838–0.895), and a Youden index of 0.590 (95% CI, 0.480–0.694). These results confirm the model’s ability to maintain reliable diagnostic utility across chronologically distinct patient presentations.

Furthermore, the models exhibited certain diagnostic characteristics that may be relevant to ED workflows. High specificity remained a consistent advantage across the XGB, LDA, and MLP models (> 90%), providing strong confidence in correctly identifying nonintoxicated patients and minimizing false alarms. Alongside this high specificity, the XGB model achieved the highest F1 score (0.658) and area under the precision-recall curve (0.681, 0.576–0.776) among the evaluated algorithms. This relatively balanced precision-recall performance suggests that the XGB model has the potential to maintain diagnostic stability even when managing the imbalanced prevalence of positive cases typically encountered in emergency settings. Notably, however, the moderate sensitivity observed across the models (0.389–0.708) indicates that these algorithms are not optimized as primary rule-out tools, as they may fail to capture all positive instances.

### Feature Interpretation

The feature importance and interactions were visualized via Shapley additive explanations analysis (Figure 3). Positive urine immunoassay results for amphetamine and ketamine, along with a self-reported history of stimulant use, were identified as the most influential predictors. The analysis highlighted an age-related pattern, with younger individuals showing a greater likelihood of psychostimulant intoxication. Clinically, the model’s predictions were influenced by a cluster of neuropsychiatric symptoms, including bizarre behavior, hallucination, and mental excitation. Additionally, tachycardia and elevated creatinine concentrations were linked to increased prediction of psychostimulant intoxication. Interestingly, high systolic blood pressure did not always correlate with increased prediction probability, suggesting a complex relationship between physiological factors and psychostimulant intoxication.

### Physician Performance Evaluation and Secondary Outcome

Table 3 summarizes the performance of the five physicians. Human evaluation was notably experience-dependent; while the toxicologist provided the most balanced clinical results (Youden index, 0.500), senior physicians prioritized specificity to minimize false-positive results. Regarding the secondary outcome, the XGB model outperformed junior physicians in both specificity (0.910 vs 0.633–0.867) and sensitivity (0.681 vs 0.500–0.633). Its specificity was also comparable to that of senior physicians (0.900–0.933). Notably, the toxicologist’s sensitivity (0.767) remained higher than all ML models, highlighting the essential role of specialist consultation in differentiating ambiguous or atypical cases.

## DISCUSSION

In this multicenter study, we demonstrated that ML algorithms provide reliable diagnostic utility for identifying psychostimulant intoxication among ED patients suspected of substance use. Our findings confirmed the primary hypothesis, because the models—particularly the XGB algorithm—maintained consistent diagnostic discrimination and overall accuracy across the temporal validation cohort. Furthermore, the study satisfied the secondary outcome by demonstrating that the algorithm’s performance is comparable to that of experienced physicians. Specifically, the XGB model outperformed junior physicians in both sensitivity and specificity. These results suggest that ML can serve as a standardized diagnostic benchmark and a reliable second opinion, potentially reducing clinical variability and mitigating diagnostic anchoring in fast-paced emergency settings.

Decision tree-based methods may achieve strong performance in structured clinical datasets because of their ability to handle complex nonlinear relationships and feature interactions, which are common in clinical data. Previous studies have demonstrated that tree-based ensemble methods often perform competitively or outperform deep learning approaches on tabular datasets.^17^ These models are particularly effective for heterogeneous data, such as age, vital signs, toxidromes, and laboratory results, as they can automatically capture intricate patterns without extensive preprocessing, such as scaling or normalization. In addition, tree-based methods are relatively robust to noisy observations, and several tree-based algorithms incorporate built-in mechanisms for handling incomplete observations, making them well suited for real-world clinical datasets.^18^,^19^

The diagnostic profile of our models, characterized by high specificity and moderate sensitivity, reflects a clinically appropriate trade-off for the ED. This balance arises from two key considerations. First, there is a distinction between detecting drug exposure and identifying clinically significant intoxication. Our reference standard, LC-MS/MS, uses a sensitive cutoff (50 ng/mL) to detect exposure regardless of clinical presentation, whereas the ML models rely on clinical features that are more evident during active intoxication. Because most test-positive cases in our cohort were clinically mild and safely discharged, the moderate sensitivity likely reflects limited detection of subclinical exposures without meaningful physiologic disturbance. Second, prioritizing specificity helps mitigate diagnostic anchoring and alarm fatigue in the ED. Overly sensitive models may increase false positives and lead to premature attribution of symptoms to drug intoxication.

In contrast, a highly specific model supports confident “rule-in” decisions when positive, while negative predictions encourage clinicians to maintain a broad differential diagnosis and actively evaluate other critical conditions, such as central nervous system infection, intracranial hemorrhage, or sepsis. When comparing physician performance to algorithmic models, we observed complementary roles between ML algorithms and clinical experts. The XGB model demonstrated an ability to mitigate experience-related variability by achieving diagnostic specificity and precision comparable to those of senior emergency physicians. This suggests that ML models can provide a standardized second opinion for junior clinicians. Additionally, toxicologist consultation remains vital for the management of complex or atypical cases. This synergy underscores the potential to integrate ML with clinical expertise to enhance diagnostic stability and support decision-making in fast-paced settings.^20^

The inclusion of ELISA results in the dataset addresses real-world diagnostic challenges in emergency settings. As shown in Supplementary Table S2, half of the psychostimulant-positive patients had mixed substance exposure, and discrepancies were observed, with 30 amphetamine-positive and 14 ketamine-positive patients in the nonpsychostimulant group, according to LC-MS/MS. These findings highlight the uncertainty of relying solely on ELISA results. By analyzing large datasets and recognizing complex patterns,^7^,^21^ ML can leverage these discrepancies to increase diagnostic precision.

The practical utility of our model lies in its integration into the existing clinical workflow during the management stage of model development.^22^ We propose a pathway where the algorithm is embedded directly into the electronic health record, using structured history, physical examination data, and automatically integrated laboratory values. To optimize clinician acceptance and mitigate alert fatigue, we recommend an “on-demand” trigger mechanism. By requiring the physician to manually initiate the prediction when substance use is suspected, the system provides targeted insights without increasing the overall cognitive load or inducing unnecessary warnings. The tool is strategically deployed at a critical “diagnostic touchpoint”^23^, specifically immediately following the initial physician evaluation. This timing is essential because it captures the window where clinical uncertainty is highest: foundational data (eg, vitals and chemistry) are accessible, but gold-standard confirmatory results (eg, LC-MS/MS) remain pending. Given the compressed time frames inherent to the ED, this automated “second opinion” serves as a vital safeguard during the high-stakes period before definitive results are available.

In practice, the model functions as a digital second opinion designed to prevent diagnostic anchoring—a common hurdle in the fast-paced environment of the ED. A positive prediction provides the clinician with the diagnostic confidence to promptly initiate critical interventions, such as sedation, supportive care, and aggressive cooling for hyperthermia, while simultaneously triggering toxicologist consultation and confirmatory testing. Conversely, a negative prediction encourages the physician to broaden the differential diagnosis to include medical mimics such as sepsis, encephalitis, thyrotoxicosis, or primary psychiatric disorders. By providing evidence-based reinforcement at a critical diagnostic touchpoint,^23^ the tool enhances the precision of emergency care and ensures that critical management pathways are activated or ruled out with greater statistical certainty.

## LIMITATIONS

Our study has several limitations. First, the 110-substance panel used may not encompass the full range of psychostimulants in the evolving unregulated substance market, potentially missing newer substances. Second, the LC-MS/MS results were reported in a qualitative format, which limits the ability to investigate the relationship between varying concentrations (trace, low, moderate, or high) and clinical symptoms.

Third, while efforts such as feature selection, decision tree pruning, and data balancing via SMOTE were employed, the models presented a common ML challenge of reduced performance from training to testing datasets.^24^,^25^ Strategies to enhance model generalizability could include expanding the training dataset, incorporating additional features such as wearable device data on tremors, posture, or facial expressions, and employing a physician feedback mechanism.^26^ Fourth, physicians reviewed medical charts retrospectively, which may have led to the loss of critical clinical information such as diagnostic intuition, facial expressions, posture, and tone of speech. The absence of these clues might have influenced their performance.

Finally, our study is limited by the absence of external geographic validation. Although the model was developed and internally validated using a multicenter dataset, its predictive logic is inherently influenced by the clinical practice patterns and laboratory ordering behaviors specific to the Taiwan healthcare system. For example, institutional thresholds for ordering diagnostic tests (eg, brain CT or certain biochemical markers) are not globally uniform. This leads to informative missingness, where the absence of a test reflects a clinician’s judgment of low risk; a model trained on such patterns may require recalibration before application in different diagnostic cultures. Furthermore, regional substance exposure patterns are highly volatile and subject to rapid shifts in unregulated drug markets. Because our cohort reflects substance prevalence in Taiwan from 2019–2020, our findings may not immediately generalize to other regions or timeframes. Future research using international cohorts and federated learning frameworks^27^ will be essential to validate the model’s performance against diverse, site-specific practice protocols and shifting epidemiological trends.

## CONCLUSION

This multicenter study demonstrated that ML algorithms provide reliable diagnostic utility for identifying psychostimulant intoxication among ED patients with suspected substance use. While their moderate sensitivity precludes their use as standalone rule-out instruments, their high specificity offers a standardized confirmatory benchmark that performs comparably to senior emergency physicians.

## Supplementary Information







## Figures and Tables

**Figure 1:**
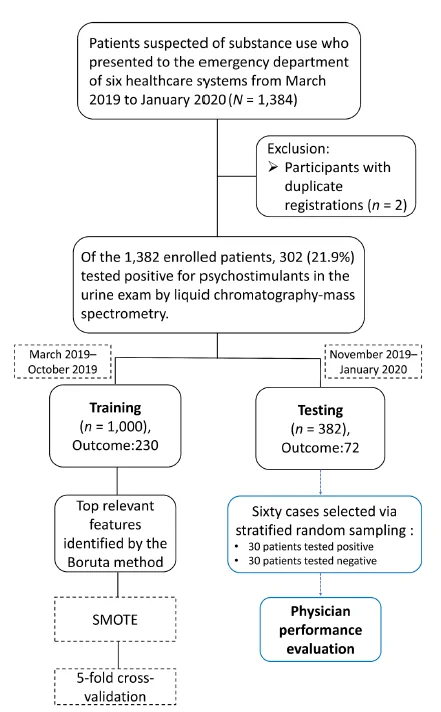
Flowchart of patient enrollment, temporal validation strategy, and machine learning development pipeline for predicting psychostimulant intoxication in emergency department patients. This study employs a temporal split to validate the algorithm’s diagnostic stability across chronologically distinct cohorts. The training dataset development incorporated the Boruta method for feature selection, the synthetic minority oversampling technique to address class imbalance, and 5-fold cross-validation for internal validation. Finally, 60 cases were randomly selected from the testing database for a comparative assessment of physician performance. *SMOTE*, synthetic minority oversampling technique.

**Figure 2:**
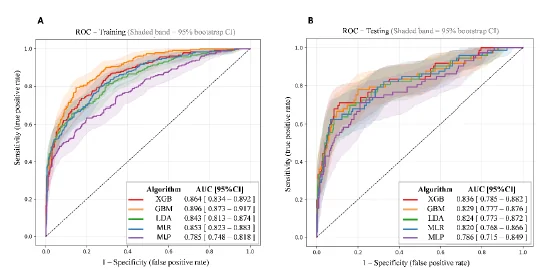
Receiver operating characteristic curves for the machine learning algorithms for predicting psychostimulant intoxication. Using the top 20 features identified by the Boruta method, all five algorithms demonstrated robust performance on the training dataset, with areas under the curve (AUC) ranging from 0.785 to 0.896 (**A**). When applied to the temporal testing dataset, the models maintained consistent diagnostic utility, achieving AUC ranging from 0.786 to 0.836 (**B**). *AUC*, area under the curve; *GBM*, gradient boosting machine; *LDA*, linear discriminant analysis; *MLP*, multilayer perceptron; *MLR*, multivariable logistic regression; *ROC*, receiver operating characteristic; *XGB*, extreme gradient boosting.

**Figure 3:**
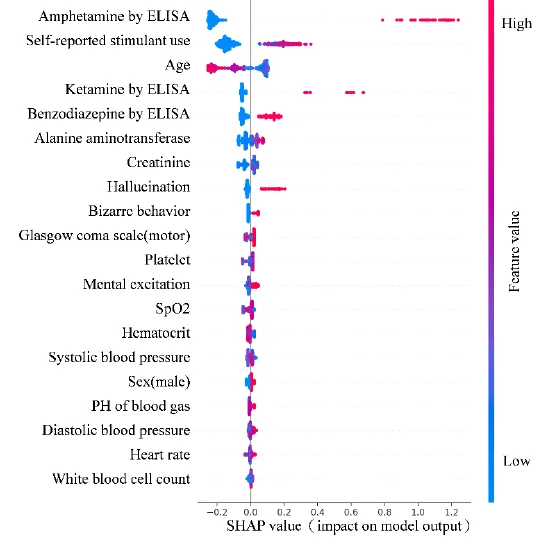
Shapley additive explanations analysis of the top 20 features in the gradient boosting machines model for predicting psychostimulant intoxication. Features are ranked in order of importance, with the color gradient indicating feature values (red for higher values, blue for lower values). Eg, in the feature “self-reported stimulant use,” high values (red dots) are associated with high SHAP values, indicating a strong predictive impact on psychostimulant intoxication. For the feature “age,” younger age (blue dots) was more predictive of psychoactive stimulant exposure (high SHAP value). *ELISA*, enzyme-linked immunosorbent *assay; SHAP, Shapley additive explanations.*

**Table 1:** Comparative analysis of demographic data, variable characteristics, laboratory results, and emergency department disposition between the psychostimulant intoxication and nonintoxication groups among patients with suspected substance use.

Feature Variables	Nonintoxication (*n* = 1,080)	Psychostimulant intoxication (*n* = 302)	Total (*N* = 1,382)	Missing Data
	Mean (SD) / *n* (%)	Mean (SD) / *n* (%)	Mean (SD) / *n* (%)	*n* (%)
Age (years)*	45.7 (20.0)	37.0 (14.0)	43.8 (19.2)	2 (0.1)$
Male sex*	572 (53.0)	193 (63.9)	765 (55.3)	0 (0)
Body temperature (°C)*	36.6 (1.0)	36.8 (1.2)	36.7 (1.0)	3 (0.2)$
Heart rate (per min)*	96.4 (24.3)	106.8 (27.3)	98.7 (25.3)	4 (0.3)$
Respiratory rate (per min)	19.7 (3.4)	19.7 (3.22)	19.7 (3.3)	6 (0.4)$
Systolic blood pressure (mm Hg)	130.2 (29.7)	127.4 (28.0)	129.6 (29.4)	9 (0.7)$
Diastolic blood pressure (mm Hg)	80.0 (19.7)	79.0 (20.4)	79.8 (19.9)	9 (0.7)$
Mental status*				0 (0)
Awake	376 (34.8)	78 (25.8)	454 (32.9)	
Depression	514 (47.5)	100 (33.1)	614 (44.4)	
Excitation	190 (17.6)	124 (41.1)	314 (22.7)	
Bizarre behavior or speech*	213 (19.7)	124 (41.1)	337 (24.3)	0 (0)
Hallucination*	64 (5.9)	66 (21.9)	130 (9.4)	0 (0)
Agitation*	190 (17.6)	124 (41.1)	314 (22.7)	0 (0)
Palpitation*	35 (3.2)	19 (6.3)	54 (3.9)	0 (0)
Self-reported substance use*	88 (8.1)	96 (31.8)	184 (13.3)	0 (0)
Violent behavior	153 (14.1)	50 (16.6)	203 (14.7)	0 (0)
Self-harm	248 (22.9)	83 (27.5)	331 (23.9)	0 (0)
Chest pain	51 (4.7)	14 (4.6)	65 (4.7)	0 (0)
Dyspnea	100 (9.2)	27 (8.9)	127 (9.2)	0 (0)
Nausea/vomiting	94 (8.7)	17 (5.6)	111 (8.0)	0 (0)
Congestive heart failure	35 (3.2)	5 (1.7)	40 (2.9)	0 (0)
Chronic lung disease	13 (1.2)	3 (1.0)	16 (1.2)	0 (0)
End-stage renal disease	19 (1.8)	2 (0.7)	21 (1.5)	0 (0)
Liver cirrhosis	43 (4.0)	5 (1.7)	48 (3.5)	0 (0)
Immunocompromised state*	31 (2.9)	16 (5.3)	47 (3.4)	0 (0)
Glasgow Coma Scale*				5 (0.4)
GCS 15	376 (34.8)	78 (25.8)	454 (32.9)	
Mild (GCS 13–14)	313 (28.9)	120 (39.7)	433 (31.3)	
Moderate (GCS 9–12)	155 (14.3)	49 (16.2)	204 (14.7)	
Severe (GCS 3–8)	236 (21.8)	55 (18.2)	291 (21.0)	
Pupils	627 (58.1)	184 (60.9)	811 (58.7)	0 (0)
1 mm	28 (2.6)	10 (3.3)	38 (2.8)	
2–4 mm	572 (53.0)	158 (52.3)	730 (52.8)	
≥5 mm	27 (2.5)	16 (5.3)	43 (3.1)	
Breathing sounds (abnormal)*	84 (7.8)	13 (4.3)	97(7.1)	0 (0)
Bowel sounds (hyperactive)	18 (1.7)	9 (3.0)	27 (2.0)	0 (0)
Urinary incontinence	58 (5.4)	12 (4.0)	70 (5.1)	0 (0)
Facial flush	72 (6.7)	15 (5.0)	87 (6.3)	0 (0)
Diaphoresis	57 (5.3)	24 (8.0)	81 (5.9)	0 (0)
Seizure (first time)	106 (9.8)	21 (7.0)	127 (9.2)	0 (0)
Tremor	61 (5.6)	16 (5.3)	77 (5.6)	0 (0)
Myoclonus/rigidity	62 (5.7)	13 (4.3)	75 (5.4)	0 (0)
Computed tomography of brain*	492 (45.6)	124 (41.1)	616 (44.5)	766 (55.4)†
Normal	371 (34.4)	104 (34.4)	475 (34.3)	
Intracranial hemorrhage	58 (5.4)	4 (1.3)	62 (4.5)	
Other findings	63 (5.8)	16 (5.3)	79 (5.7)	
SpO_2_ (%)	95.3 (8.9)	95.8 (5.3)	95.4 (8.1)	227 (16.4)$
White blood cell count (× 10^3^)*	10.3 (5.4)	11.5 (6.9)	10.6 (5.8)	68 (4.9)$
Hematocrit (%)*	39.4 (6.3)	40.8 (5.7)	39.7 (6.2)	109 (7.9)$
Platelet count (× 10^3^)*	249.8 (82.0)	272.5 (96.3)	254.7 (85.8)	73 (5.3)$
Creatinine (mg/dL)	1.2 (1.1)	1.3 (1.7)	1.2 (1.2)	77 (5.6)$
Sodium (mEq/L)	138.1 (5.7)	138.1 (4.6)	138.1 (5.5)	77 (5.6)$
Potassium (mEq/L)	3.8 (0.6)	3.8 (0.6)	3.78 (0.6)	73 (5.3)$
Blood glucose (mg/dL)	141 (96.4)	134 (122)	140 (103)	212 (15.3)$
Creatine kinase > 1,000 (U/L)*	287 (26.5)	38 (12.6)	325 (23.5)	913 (66.0)†
Alanine aminotransferase (IU/L)	47.7 (198)	78.3 (392)	54.4 (253)	234 (16.9)$
Lactate (mmol/L)	2.20 (3.12)	2.31 (3.25)	2.22 (3.15)	781 (56.4)$
Elevated Troponin I	97 (9.0)	31 (10.3)	128 (9.3)	757 (54.7)†
Blood alcohol concentration >10 mg/dL	116 (10.7)	24 (8.0)	140 (10.1)	818 (59.1)†
pH of blood gas	7.36 (0.09)	7.37 (0.08)	7.36 (0.09)	471 (34.0)$
pCO_2_ of blood gas (mm Hg)	42.2 (9.7)	41.0 (8.4)	41.9 (9.4)	471 (34.0)$
HCO_3_ of blood gas (mmol/L)	22.9 (4.3)	22.7 (4.2)	22.8 (4.3)	471 (34.0)$
Amphetamine by ELISA*	30 (2.8)	111 (36.8)	141 (10.2)	60 (4.3)
Morphine by ELISA*	56 (5.2)	36 (11.9)	92 (6.7)	60 (4.3)
Ketamine by ELISA*	14 (1.3)	59 (19.5)	73 (5.3)	99 (7.2)
MDMA by ELISA*	2 (0.2)	11 (3.6)	13 (0.9)	99 (7.2)
Benzodiazepine by ELISA*	226 (20.9)	98 (32.5)	324 (23.4)	60 (4.3)
Cannabinoids by ELISA	10 (0.9)	3 (1.0)	13 (0.9)	121 (8.7)
ED disposition				0 (0)
Discharged home	590 (54.6)	180 (59.6)	770 (55.7)	
Admitted to ward	297 (27.5)	72 (23.8)	369 (26.7)	
Admitted to ICU	185 (17.1)	46 (15.2)	231 (16.7)	
Death	8 (0.7)	4 (1.3)	12 (0.9)	

# The independent *t*-test was used for continuous variables and the chi-square test was used for categorical variables.

* A *P* value < .05 was deemed statistically significant.

$ Mean imputation.

† Dummy coding for informative missingness (negative vs not performed)

GCS scores: mode imputation. Other categorical variables were inherently binary based on the structured medical records.

*ED*, emergency department; *ELISA*, enzyme-linked immunosorbent assay; *GCS*, Glasgow Coma Scale; *ICU*, intensive care unit; *MDMA*, 3–4 methylenedioxymethamphetamine; *SD*, standard deviation.

**Table 2:** Model prediction performance for identifying psychostimulant intoxication in the training and testing datasets using top 20 features selected by Boruta method. Diagnostic accuracies for the extreme gradient boosting, multilayer perceptron, and linear discriminant analysis models exceeded 86%. High specificity was a consistent strength, surpassing 90% in the multilayer perceptron (0.958), linear discriminant analysis (0.935), and extreme gradient boosting (0.910) models, underscoring their effectiveness in correctly identifying negative cases. However, diagnostic sensitivity ranged from 0.389 to 0.708, indicating that the models may fail to identify approximately 29% to 61% of positive cases. The Youden index identified the extreme gradient boosting model (0.590) as providing the most effective balance for clinical application.

Performance Metric	Training					Testing				
	XGB	GBM	LDA	MLR	MLP	XGB	GBM	LDA	MLR	MLP
AUC [95% CI]	0.864 [0.834 – 0.892]	0.896 [0.873 – 0.917]	0.843 [0.813 – 0.874]	0.853 [0.823 – 0.883]	0.785 [0.748 – 0.818]	0.836 [0.785 – 0.882]	0.829 [0.777 – 0.876]	0.824 [0.773 – 0.872]	0.820 [0.768 – 0.866]	0.786 [0.715 – 0.849]
Accuracy [95% CI]	0.844 [0.820 – 0.866]	0.834 [0.812 – 0.858]	0.858 [0.838 – 0.879]	0.788 [0.762 – 0.812]	0.836 [0.813 – 0.858]	0.866 [0.838 – 0.895]	0.798 [0.764 – 0.832]	0.864 [0.835 – 0.893]	0.764 [0.728 – 0.801]	0.851 [0.814 – 0.885]
Specificity	0.910 [0.889 – 0.929]	0.853 [0.830 – 0.879]	0.968 [0.955 – 0.979]	0.818 [0.789 – 0.844]	0.974 [0.962 – 0.984]	0.910 [0.882 – 0.935]	0.819 [0.783 – 0.854]	0.935 [0.914 – 0.958]	0.784 [0.745 – 0.822]	0.958 [0.935 – 0.978]
Sensitivity (recall)	0.622 [0.559 – 0.686]	0.770 [0.716 – 0.822]	0.491 [0.430 – 0.554]	0.686 [0.628 – 0.748]	0.374 [0.313 – 0.435]	0.681 [0.589 – 0.767]	0.708 [0.616 – 0.794]	0.556 [0.460 – 0.651]	0.681 [0.588 – 0.768]	0.389 [0.273 – 0.507]
PPV (precision)	0.675 [0.609 – 0.734]	0.610 [0.553 – 0.665]	0.819 [0.746 – 0.874]	0.530 [0.474 – 0.586]	0.811 [0.726 – 0.874]	0.636 [0.526–0.735]	0.477 [0.384 – 0.571]	0.667 [0.541 – 0.776]	0.422 [0.335 – 0.513]	0.683 [0.550 – 0.790]
NPV	0.889 [0.866 – 0.910]	0.925 [0.904 – 0.942]	0.864 [0.840–0.886]	0.897 [0.873 – 0.918]	0.839 [0.813 – 0.862]	0.924 [0.888 – 0.951]	0.924 [0.887 – 0.949]	0.901 [0.865 – 0.930]	0.914 [0.874 – 0.942]	0.871 [0.835 – 0.905]
PRC	0.729 [0.680 – 0.779]	0.759 [0.704 – 0.808]	0.719 [0.669 –0.773]	0.717 [0.665 – 0.774]	0.633 [0.571 – 0.692]	0.681 [0.576 – 0.776]	0.658 [0.550 – 0.762]	0.659 [0.555 – 0.757]	0.623 [0.516 – 0.737]	0.588 [0.468 – 0.701]
F1 Score	0.647 [0.593 – 0.698]	0.681 [0.633 – 0.725]	0.614 [0.553 – 0.672]	0.598 [0.547 – 0.646]	0.512 [0.444 – 0.575]	0.658 [0.585 – 0.731]	0.570 [0.500–0.637]	0.606 [0.534 – 0.677]	0.521 [0.453 – 0.588]	0.496 [0.425 – 0.565]
Youden index	0.532 [0.465 – 0.598]	0.623 [0.562 – 0.682]	0.459 [0.393 – 0.526]	0.505 [0.438 – 0.570]	0.348 [0.285 – 0.411]	0.590 [0.480 – 0.694]	0.528 [0.425 – 0.642]	0.491 [0.395 – 0.601]	0.464 [0.355 – 0.564]	0.347 [0.285 – 0.415]

*AUC*, area under the receiver operating characteristics curve; *GBM*, gradient boosting machine; *LDA*, linear discriminant analysis; *MLP*, multilayer perceptron; *MLR*, multivariate logistic regression; *NPV*, negative predictive value; *PPV*, positive predictive value; *PRC*, area under the precision-recall curve; *XGB*, extreme gradient boosting.

**Table 3:** Diagnostic performance of physicians in identifying psychostimulant intoxication on a 60-case subset.

Performance Metric	Physician performance evaluation				
	Toxicologist	Senior emergency physician 1	Senior emergency physician 2	Junior emergency physician 1	Junior emergency physician 2
Accuracy	0.750	0.683	0.667	0.683	0.633
Specificity	0.733	0.900	0.933	0.867	0.633
Sensitivity (recall)	0.767	0.467	0.400	0.500	0.633
PPV (precision)	0.742	0.824	0.857	0.789	0.633
NPV	0.759	0.628	0.609	0.634	0.633
Youden index	0.500	0.367	0.333	0.367	0.266

*NPV*, negative predictive value; *PPV*, positive predictive value.
