# Supplementary material for: Machine Learning Algorithms for Predicting Psychostimulant Intoxication in Emergency Department Patients: A Multicenter Study
Source: West J Emerg Med. 2026 Oct 18;27(6):1599–610. doi: 10.5811/westjem.63917 (PMC13761782; doi:10.5811/westjem.63917)
Supplement: Supplementary file 1 [file wjem-27-1599-s001.docx]

**Supplementary Table S1.** List of 110 drugs and metabolites detected by liquid chromatography-mass spectrometry for patients with suspected illicit substance intoxication. Of these 110 illicit substances, 104 are classified as psychostimulants. The remaining six, marked with an asterisk (*), are categorized as non-psychostimulants. Psychostimulants are further divided into traditional psychostimulants and new psychoactive substances. The latter are defined by the United Nations Office on Drugs and Crime as those not controlled under the 1961 Single Convention on Narcotic Drugs or the 1971 Convention on Psychotropic Substances but potentially posing a public health threat. The cutoff concentration for urine confirmatory testing using liquid chromatography-mass spectrometry is set at 50 ng/mL.

| **Psychostimulant NPS** | | | |
| --- | --- | --- | --- |
| **Drug types** | **Compounds** | | |
| Synthetic Cannabinoids | JWH-018^1^  JWH-019^2^  JWH-022^3^  JWH-073^4^  JWH-081^5^  JWH-203^6^ | JWH-250^7^  NM-2201^8^  UR-144^9^  XLR-11^10^  APINACA^11^  MDMB-CHMICA^12^ | AB-FUBINACA^13^  AB-PINACA^14^  AMB-FUBINACA^15^  AB-CHMINACA^16^  5-Chloro-UR-144^17^ |
| Phenethylamine | 2C-C^18^  2C-I^19^  2C-E^20^  5-MAPB^21^  5-MAPDB^22^  5-APDB^23^  MDDMA^24^  MBDB^25^ | 6-methoxy methylone  N-Hydroxy-MDA^26^  25I-NBOMe^27^  25B-NBOMe^28^  4-Bromoamphetamine  4-Fluoroamphetamine  4-Chloroamphetamine | 4-Fluoromethamphetamine  4-Chloromethamphetamine  4-Methoxymethamphetamine  (MMA)  4-Methoxyethylamphetamine  (PMEA)  N, N-Dimethylamphetamine |
| Synthetic cathinones | Ethylone  Butylone  Pentylone  Methylone  Mephedrone  Methedrone  Benzedrone  Ephylone  Methcathinone  Eutylone  D-Tertylone MDPV^29^  TH-PVP^30^  α-PPP^31^  α-PVP^32^  α-PHPP (PV8)^33^ | α-PNP (PV10)^34^  3,4-MDPBP^35^  3,4-MDPHP^36^  4'-Methyl-α-PHP^37^  4-Chloro-α-PVP^38^  4-fluoro-α-PHP^39^  4-fluoro-α-PVP^40^  4-Methylpentedrone  Dimethylone  (bk-MDDMA)  Dibutylone  (bk-DMBDB)  4-Methylethcathinone  (4-MEC)  4-Chloroethcathinone  (4-CEC) | α-Ethylaminohexanophenone  3,4-Dimethylmethcathinone  N-Acetyl-3,4-MDMC^41^  4-Cl-N,N-DMC^42^  4-Chloromethcathinone  (4-CMC)  4-Methyl-α-ethylamino-pentiophenone (4-MEAP)  4-Bromoethcathinone  (4-BEC)  4-Bromomethcathinone  (4-BMC)  4-Ethylmethylcathinone  (4-EMC)  4-Fluoromethcathinone  (4-FMC) |
| Phencyclidine-type substances | Ketamine  Norketamine | Methoxetamine  Deschloroketamine | 4-MeO-PCP^43^  Deschloro-N-ethyl-ketamine |
| Tryptamines | Bufotenin  5-MeO-DIPT^44^  5-MeO-MiPT^45^ | 5-MeO-DMT^46^  5-MeO-DALT^47^ | α-Methyltryptamine (AMT)  N-Methyltryptamine (NMT) |
| Piperazines | BZP^48^  MBZP^49^  MT-45^50^ | DBZP^51^  TFMPP^52^ | p-Fluorophenylpiperazine  meta-Chlorophenylpiperazine |
| Other stimulants | Methiopropamine | 2-Benzhydrylpyrrolidine  (Desoxy-D2PM) | 2-MAPB^53^ |
| **Traditional Psychostimulants** | | | |
| **Drug types** | **Compounds** | | |
| Amphetamine-type stimulants^†^ | Amphetamine Methamphetamine  MDA^26^ MDMA^55^ | | |
| Marijuana | 11-Nor-9-carboxy-THC^54^ | | |
| Cocaine | Benzoylecgonine | | |
| **Non-psychostimulant NPS** | | | |
| **Drug types** | **Compounds** |  |  |
| Benzodiazepines^‡^ | Etizolam* | Phenazepam* | Diclazepam* |
| **Non-psychostimulants that Classified as Traditional Illicit Substances** | | | |
| **Drug types** | **Compounds** |  |  |
| Opioids | Codeine* | Morphine* | 6-Acetylmorphine* |

† The LC-MS/MS assay does not differentiate between the d- and l-isomers of amphetamine, reporting a combined signal.

‡ Commonly prescribed medical benzodiazepines (eg, diazepam, alprazolam, lorazepam, temazepam) are not included in the LC-MS/MS panel.

*LC-MS/MS,* liquid chromatography-mass spectrometry/mass spectrometry*; NPS*, new psychoactive substances.

1.	JWH-018: 1-pentyl-3-(1-naphthoyl)indole

2.	JWH-019: (1-hexyl-1H-indol-3-yl)-1-naphthalenyl-methanone

3.	JWH-022: 1-naphthalenyl[1-(4-penten-1-yl)-1H-indol-3-yl]-methanone

4.	JWH-073: (1-butyl-1H-indol-3-yl)-1-naphthalenyl-methanone

5.	JWH-081: (4-methoxy-1-naphthalenyl)(1-pentyl-1H-indol-3-yl)-methanone

6.	JWH-203: 2-(2-chlorophenyl)-1-(1-pentyl-1H-indol-3-yl)-ethanone

7.	JWH-250: 1-pentyl-3-(2-methoxyphenylacetyl) indole

8.	NM-2201: naphthalen-1-yl 1-(5-fluoropentyl)-1H-indole-3-carboxylate

9.	UR-144: (1-pentyl-1H-indol-3-yl)(2,2,3,3-tetramethylcyclopropyl)-methanone

10.	XLR-11: (1-(5-fluoropentyl)-1H-indol-3-yl)(2,2,3,3-tetramethylcyclopropyl)methanone

11.	APINACA: N-(1-Adamantyl)-1-pentyl-1H-indazole-3-carboxamide

12.	MDMB-CHMICA: N-[[1-(cyclohexylmethyl)-1H-indol-3-yl]carbonyl]-3-methyl-L-valine, methyl ester

13.	AB-FUBINACA: N-[(1S)-1-(aminocarbonyl)-2-methylpropyl]-1-[(4-fluorophenyl)methyl]-1H-indazole-3-carboxamide

14.	AB-PINACA: (S)-N-(1-amino-3-methyl-1-oxobutan-2-yl)-1-pentyl-1H-indazole-3-carboxamide

15.	AMB-FUBINACA: N-[[1-[(4-fluorophenyl)methyl]-1H-indazol-3-yl]carbonyl]-L-valine, methyl ester

16.	AB-CHMINACA: N-[(1S)-1-(aminocarbonyl)-2-methylpropyl]-1-(cyclohexylmethyl)-1H-indazole-3-carboxamide

17.	5-Chloro-UR-144: (1-(5-chloropentyl)-1H-indol-3-yl)(2,2,3,3-tetramethylcyclopropyl)methanone

18.	2C-C: 2,5-dimethoxy-4-chlorophenethylamine

19.	2C-I: 2,5-dimethoxy-4-iodophenethylamine

20.	2C-E: 2,5-dimethoxy-4-ethylphenethylamine

21.	5-MAPB: 1-(benzofuran-5-yl)-N-methylpropan-2-amine

22.	5-MAPDB: 1-(2,3-dihydrobenzofuran-5-yl)-N-methylpropan-2-amine

23.	5-APDB: 2,3-dihydro-α-methyl-5-benzofuranethanamine

24.	MDDMA: N,N,α-trimethyl-1,3-benzodioxole-5-ethanamine

25.	MBDB: α-ethyl-N-methyl-1,3-benzodioxole-5-ethanamine

26.	MDA: 3,4-methylenedioxyamphetamine

27.	25I-NBOMe: 2-(4-iodo-2,5-dimethoxyphenyl)-N-[(2-methoxyphenyl)methyl]ethanamine

28.	25B-NBOMe: 4-bromo-2,5-dimethoxy-N-[(2-methoxyphenyl)methyl]-benzeneethanamine

29.	MDPV: methylenedioxypyrovalerone

30.	TH-PVP: 2-(pyrrolidin-1-yl)-1-(5,6,7,8-tetrahydronaphthalen-2-yl)pentan-1-one

31.	α-PPP: α-pyrrolidinopropiophenone

32.	α-PVP: α-pyrrolidinopentiophenone

33.	α-PHPP (PV8): 1-phenyl-2-(1-pyrrolidinyl)-1-heptanone monohydrochloride

34.	α-PNP(PV-10): 1-phenyl-2-(1-pyrrolidinyl)-1-nonanone

35.	3,4-MDPBP: 3',4'-methylenedioxy-α-pyrrolidinobutiophenone

36.	3,4-MDPHP: 3',4'-methylenedioxy-α-pyrrolidinohexiophenone

37.	4'-Methyl-α-PHP: 2-(pyrrolidin-1-yl)-1-(p-tolyl)hexan-1-one

38.	4-Chloro-α-PVP: 1-(4-chlorophenyl)-2-(1-pyrrolidinyl)-1-pentanone

39.	4-fluoro-PHP: 1-(4-fluorophenyl)-2-(pyrrolidin-1-yl)hexan-1-one

40.	4-fluoro-α-PVP: 1-(4-fluorophenyl)-2-(1-pyrrolidinyl)-1-pentanone

41.	N-Acetyl-3,4-MDMC: N-acetyl-3,4-methylenedioxymethcathinone

42.	4-Cl-N,N-DMC: 4-chloro-N,N-dimethylcathinone

43.	4-MeO-PCP: 1-[1-(4-methoxyphenyl)cyclohexyl]-piperidine

44.	5-MeO-DIPT: 5-methoxy-N,N-diisopropyltryptamine

45.	5-MeO-MiPT: 5-methoxy-N-methyl-N-isopropyltryptamine

46.	5-MeO-DMT: 5-methoxy-N,N-dimethyltryptamine

47.	5-MeO-DALT: N,N-di allyl-5-methoxy tryptamine

48.	BZP: 1-(phenylmethyl)-piperazine

49.	MBZP: 1-methyl-4-(phenylmethyl)-piperazine

50.	MT-45: 1-cyclohexyl-4-(1,2-diphenylethyl)-piperazine

51.	DBZP: dibenzylpiperazine

52.	TFMPP: trifluoromethylphenylpiperazine

53.	2-MAPB: N,α-dimethyl-2-benzofuranethanamine

54.	THC: tetrahydrocannabinol

55.	MDMA: 3,4-methylenedioxymethamphetamine
