# Supplementary material for: Machine Learning Algorithms for Predicting Psychostimulant Intoxication in Emergency Department Patients: A Multicenter Study
Source: West J Emerg Med. 2026 Oct 18;27(6):1599–610. doi: 10.5811/westjem.63917 (PMC13761782; doi:10.5811/westjem.63917)
Supplement: Supplementary file 2 [file wjem-27-1599-s002.docx]

**Supplementary Figure S1.** The categories of substances detected by liquid chromatography-mass spectrometer among patients suspected of illicit substance intoxication.

* This item contains both amphetamine and methamphetamine.

*MDA*, 3,4-methylenedioxyamphetamine; *MDMA*, 3,4-methylenedioxymethamphetamine.

* This item contains both amphetamine and methamphetamine.

*MDA*, 3,4-methylenedioxyamphetamine; *MDMA*, 3,4-methylenedioxymethamphetamine.
