# Supplementary material for: Machine Learning Algorithms for Predicting Psychostimulant Intoxication in Emergency Department Patients: A Multicenter Study
Source: West J Emerg Med. 2026 Oct 18;27(6):1599–610. doi: 10.5811/westjem.63917 (PMC13761782; doi:10.5811/westjem.63917)
Supplement: Supplementary file 3 [file wjem-27-1599-s003.docx]

| **Supplementary Table S2.** Combination patterns of substances in 1,382 enrolled patients suspected of illicit substance intoxication. | | |
| --- | --- | --- |
| **Substances Detected by LC-MS/MS*** | **Patients(n)** | **Sum (%)** |
| **Psychostimulants** |  | 302 (21.9) |
| **Traditional psychostimulants**^†^ |  | 142 (10.3) |
| (Meth)amphetamines | 46 |  |
| (Meth)amphetamines and NPS | 53 |  |
| (Meth)amphetamines and opioids | 18 |  |
| (Meth)amphetamines, NPS, and MDMA/MDA | 13 |  |
| (Meth)amphetamines and MDMA/MDA  (Meth)amphetamines and BZD*** | 1  1 |  |
| MDMA/MDA, and NPS | 7 |  |
| MDMA | 1 |  |
| THC-COOH | 1 |  |
| THC-COOH and opioid | 1 |  |
| **Psychostimulant NPS** |  | 160 (11.6) |
| One kind of psychostimulant NPS | 96 |  |
| Cathinones and ketamines | 26 |  |
| ≧2cathinones | 15 |  |
| Psychostimulant NPS and opioids  Psychostimulant NPS and BZD^‡^ | 13  6 |  |
| Other combinations | 4 |  |
| **Non-Psychostimulant exposure** |  | 1,080 (78.1) |
| LC-MS/MS negative |  | 1,012 (73.2) |
| Pure opioids |  | 68 (4.9) |
| * The classifications, definitions, and detailed items of the 110 illicit substances are shown in Supplementary Table S1.  † Traditional psychostimulants include amphetamine, methamphetamine, 3,4-methylenedioxy-methamphetamine (MDMA), 3,4-methylenedioxy-amphetamine (MDA), and cannabinoid (tetrahydrocannabinol-9-carboxylic acid, THC-COOH).  ‡ Illicit benzodiazepines include etizolam, phenazepam, and diclazepam.  *BZD*, benzodiazepine; *LC-MS/MS*, liquid chromatography-mass spectrometry; *NPS*, new psychoactive substances. | | |
